# On the utility of the trail making test in migraine with and without aura: a meta-analysis

**DOI:** 10.1186/s10194-020-01137-y

**Published:** 2020-06-03

**Authors:** Antonino Vallesi

**Affiliations:** 1 Department of Neuroscience & Padova Neuroscience Center, University of Padova, Via Giustiniani, 5, Padova, 35128 Italy; 2 Brain Imaging and Neural Dynamics Research Group, IRCCS San Camillo Hospital, Venice, 30126 Italy

**Keywords:** Executive functions, Mental flexibility, Migraine with aura, Migraine without aura, Neuropsychological assessment, Response speed

## Abstract

This meta-analytical review assesses the utility of the Trail Making Test (TMT), versions A and B, in detecting migraine-related cognitive deficits. A comprehensive literature search was performed in two electronic databases and other sources to obtain relevant studies administering TMT to migraine patients. Search terms included “migraine” and “Trail Making”. Only studies in which the TMT-A, TMT-B or both were administered to adult patients suffering from migraine with and without aura were included. All pooled meta-analyses were based on random effects models. A total of 14 studies for TMT-A and 15 for TMT-B met inclusion criteria and were subjected to meta-analyses. Results showed that performance is worse in migraine patients than in controls for both the TMT-A (Hedges’ g = −.28) and TMT-B (g = −.37), with no difference between migraine with and without aura. This study demonstrates the sensitivity of the TMT in detecting cognitive alterations in migraine. This test should be considered for inclusion in cognitive batteries assessing patients with migraine.

## Introduction

Migraine is a primary headache disorder associated with recurrent pain attacks involving throbbing or pulsing sensations, more frequently on one side of the head. Migraine attacks typically last from few hours to days, and the pain can be so incapacitating that it interferes with daily activities. These attacks could be preceded by sensory (primarily visual) disturbances called *aura* or not. While some authors have reported comparable cognitive abilities in migraineurs and healthy controls [1–5], the results of recent qualitative reviews [6, 7] suggest that, in contrast to other types of headache (e.g., tension type or cluster headache), cognitive dysfunctions are detectable in migraine sufferers even in the inter-ictal period [8], especially in clinic-based studies. These results are usually obtained above and beyond the side effects of preventive drugs and possible consequences of comorbidities, such as depression and anxiety [6]. Differences in cognitive abilities, when reported, are more often associated with migraine with aura (MwA), while whether also migraine without aura (MwoA) is related with cognitive impairment remains less clear [8].

Divergent results might also be partially due to heterogeneity of approaches used in assessing cognitive functions in individuals suffering from migraine. It would be therefore desirable to systematically assess the relevant literature. I will start by examining in this study whether migraine affects performance on the Trail Making Test (TMT), one of the most widely used neuropsychological tests to evaluate migraine-related cognitive dysfunction [9]. This test typically entails two forms: TMT-A requires patients to sequentially connect through lines 25 encircled numbers pseudo-randomly distributed on a sheet; In TMT-B patients must instead alternate between numbers and letters when connecting the different items in an ascending order (i.e., 1, A, 2, B etc.). The score of each part is calculated as the number of seconds required to complete the test. This test was incorporated into the US Army Individual Test Battery [10], and then later adapted for the Halstead-Reitan Test Battery [11–13] and other batteries [14]. The TMT-A is typically conceived as a measure of visual search and speed of processing, whereas the TMT-B is assumed to additionally measure mental flexibility and executive functions more generally [15–18]. The high popularity of the TMT in the neuropsychological assessment of cognitive and executive dysfunction in general, but also in migraine specifically, could be explained by its simplicity and short administration time.

However, to date there has not been any quantitative assessment of the sensitivity of the TMT in detecting cognitive deficits in the migraine literature. A meta-analytical approach is perfectly suited to formally test whether the TMT is sensitive and worth being used in the neuropsychological assessment of migraine, as it exhaustively reviews the literature, aggregates individual studies overcoming their limits (e.g., low power), and quantifies differences between groups.

The objective of this meta-analytical study is therefore to understand whether performance on the TMT-A and B (operationalized as the amount of time necessary to complete each form) differs between patients suffering from MwA and MwoA and matched healthy controls. The results of this quantitative review could be relevant to the clinical practitioners who want to assess cognitive disfunction in migraine and have to decide whether to include the TMT in their battery, and to generally inform the debate over whether migraine is associated with cognitive impairment or does not exert any impact on cognitive functioning [3, 5].

## Methods

### Protocol and registration

The protocol for this meta-analysis was submitted on PROSPERO (https://www.crd.york.ac.uk/PROSPERO/) with the registration ID #160041.

### Eligibility criteria

The following inclusion criteria were used to select articles for the meta-analyses: 1) Adult participants (age > 18 years) suffering from MwA or MwoA; whenever an alternative term was used in the retrieved article, that is “classic migraine” for MwA, and “common migraine” for MwoA, these were coded with the corresponding aura-related terms; 2) No comorbidity with other psychiatric/neurological conditions; 3) Testing during the inter-ictal period; 4) Inclusion of data on TMT-A, TMT-B or both; 5) Inclusion (or provision from corresponding author) of sample size for each sub-group and enough statistical information, such as means and standard deviations, and/or median and interquartile range, and/or t, F, X, so that effect sizes could be calculated or estimated; 6) Group studies (no single-cases) with a cross-sectional design; 7) Finally, given the variety of normative data available and their many limits (e.g., small sample size, restricted age and education ranges, lack of percentiles [19] cf. [18]), only articles in which TMT was also assessed in an ad-hoc matched control group were included. Studies with other types of headache, including inherited small-artery disease of the brain (CADASIL), familial hemiplegic migraine (FHM), cluster headache, and where the focus was on other pathologies were also excluded.

### Information sources

A comprehensive literature search was carried out using Pubmed and PsychInfo. References in additional articles on the topic were also checked in order to identify other possibly relevant articles. Corresponding authors or co-authors were contacted by email when statistical information was insufficient in order to obtain missing information.

### Search

The main literature search was carried out using the conjunction of the following search terms: “migraine” AND “trail making” with no restriction on publication date range. Studies should have been either published or in press to be included. All languages were considered, provided that there was an English version available. The last search was performed in the relevant databases on December first, 2019.

### Study selection

Titles and abstracts of the retrieved studies were first screened by the author to assess adhesion to eligibility criteria. Then, full texts of retrieved articles were downloaded from sources when available; otherwise a request was made to the Network Inter Library Document Exchange system (NILDE, https://nilde.bo.cnr.it/) and/or to corresponding or other authors by email. Once a full text was obtained, a further eligibility check was performed by reading the whole article.

Selection choices for some studies also deserve mention. Since only two patients out of 40 (5%) had Familiar Hemiplegic Migraine (FHE) in El-Senousy et al. [20], that study was included. In another study [21], the standard deviation was estimated using the “range” method, whereby the difference between minimum and maximum values is divided by 4 [22]. For some studies ([21, 23, 24]; and two subgroups in [25]) it was not possible to know which type of migraine patients were tested. These works were retained assuming that the majority of the recruited patients would suffer from the most common types of migraine (MwA, MwoA). One study [26] also included adolescents (age range: 15–68 years); since visual inspection of their Fig. 1 showed that a very small minority of participants were < 18 years old, that study was included. Two studies [20, 27] were assumed to focus on adults, although exact age mean/range were missing.

**Fig. 1:**
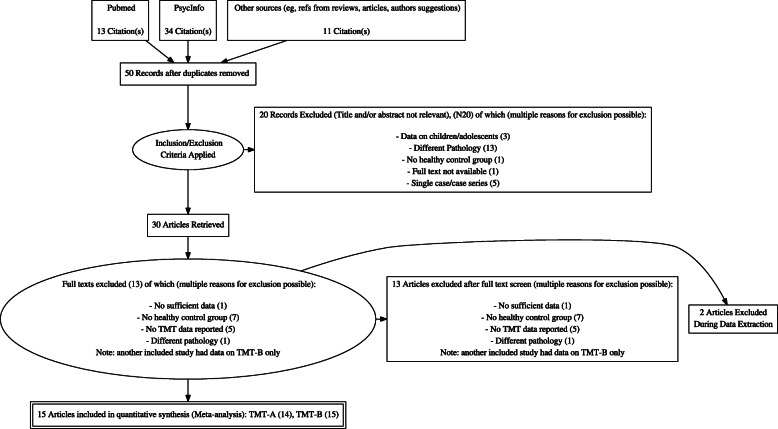
PRISMA Flow Diagram of the studied screened, assessed for eligibility and included in the review

Age and education were well-matched between migraine patients and healthy controls in the vast majority of the included studies. However, a few exceptions need to be mentioned. In Martins and colleagues [24], the control sample was significantly older than the migraine group (66.8 ± 9 vs. 61.9 ± 7.6 years). Since the direction of this age difference should have acted against the hypothesis that TMT performance is affected in migraine, we decided to keep this study in our meta-analyses. In Tessitore and associates [28], the education level was significantly higher in controls than in migraine samples (MwoA: 13.2 ± 0.64; MwA: 14.85 ± 0.55; Healthy Controls: 17.25 ± 0.4 years). Since the samples were well-matched for other demographic characteristics (age, gender) and, more crucially, since the results of all the meta-analyses remained unaffected when this study was excluded, it was kept in the analyses reported here.

### Data collection process

All statistical information necessary for performing the meta-analysis was extracted by the author from the retrieved articles, including sample size for each sub-group, and typically means and standard deviations of the number of seconds necessary to complete the TMT sub-tests or other information useful to calculate/estimate effect size. When statistical information was insufficient, the corresponding and/or another author were asked missing information by email. Data were reported, analyzed and plotted in Meta-Essentials 1.4 [29].

### Data items

The number of seconds to complete each section (TMT-A and/or TMT-B) was chosen as the dependent variable, instead of more rarely reported measures of TMT performance, such as errors, ratio (TMT-B/TMT-A) or difference (TMT-B – TMT-A) scores. Separate effect sizes were calculated for each part (A/B) of the TMT, when both were available. The migraine type in the patient sample/s was also recorded (MwA, MwoA, mixed migraine). Whether a study adopted a blind neuropsychological evaluation on TMT performance or not was also reported (if nothing was specified, the study was considered non-blinded). It was also reported whether patients were tested during the inter-ictal period, during the attack (exclusion criterion) or this information was unspecified.

### Risk of bias in individual studies

The author reported whether blinding was applied to the screened studies, to appreciate the risk of bias. To obtain more homogeneous results, studies focused on the adult age range only were included, while studies mainly recruiting children and adolescents were excluded. When reported, inclusion/exclusion criteria were noted. Since only few studies allowed patients with comorbidities or medication overuse (Table 1), their role could not be formally assessed.

**Table 1:** Summary of included studies assessing performance of patients with migraine on Trail Making Test (TMT)

Source	Recruitment	No. Patients	No. Controls	Patient description	Patients’ Age range: mean (SD)	Blind design	Inclusion criteria	TMT tests
Baschi et al. 2019 [30]	Clinic-based	21	21	MwoA	29 (4.32); Controls: 27.9 (3.16)	Yes	History of migraine of at least 5 years; at least 12 migraine attacks in the last year and < 4 attacks/month; normal brain magnetic resonance imaging, no other coexisting types of headache, absence of depression, other neurological diseases, no consumption of psychotropic drugs, including migraine prophylactics.	TMT-A & B
Burker et al. 1989 [31]	Population-based	47	24	Group 1: MwA (20); Group 2: MwoA (27)	Group 1: 19.45 (1.73); Group 2: 19.22 (1.12); Controls: 18.66 (1.09)	No	Diagnosis of common or classic migraine according to Adrasik & Burke’s criteria for diagnosing Headache (in Blumenthal & McKee, 1987).	TMT-A & B
Cai et al. 2019 [25]	Clinic-based	76	40	Sub-Group 1: Chronic migraine without medication overuse (20) Sub-Group 2: Chronic migraine with medication overuse (21) Sub-Group 3: MwoA (35)	Sub-Group 1: 48.40 (10.33); Sub-Group 2: 48.90 (13.51); Sub-Group 3: 45.89 (7.10); Controls: 47.10 (7.04)	No	Diagnosis of episodic migraine, chronic migraine with and without medication overuse headache; headache duration ≥1 year; age between 25 and 65; confirmation of nonstructural lesions according to brain CT/MRI, in the interictal periods of migraine; no headache secondary to trauma, intracranial inflammation, brain tumor, and other neurological diseases; no cerebrovascular disorders, neoplastic diseases, infectious diseases, rheumatic diseases, or connective tissue diseases; no cognitive impairment or psychiatric disease.	TMT-A & B
Calandre et al. 2002 [26]	mixed (mostly clinic-based)	60	20	Sub-Group 1: Combined (MwA, MwoA) with 20 years of illness or less Sub-Group 2: Combined (MwA, MwoA) with more than 20 years of illness	Reported age range for all groups: 15–68 years	No	Migraine history > 1 year, IQ > 80, no coexistent type of headache or concomitant organic or psychiatric disease.	TMT-A & B
Camarda et al. 2007 [32]	Clinic-based	45	90	MwoA	33.6 (8.6); Controls: 31.2 (8.2)	Yes	Migraine history ≥5 years; at least 12 migraine attacks in the last year; normal brain CT scan; absence of other coexisting types of headache; age ≤ 50 years; normal neurological examination; a minimal IQ value of 80; normal global intellectual ability; no headache attack 48 h before or after the cognitive session; no history of psychiatric disorders, seizures, head trauma, alcohol or drug abuse and cerebrovascular accident; no consumption of psychotropic drugs at the time of testing. However, MwoA showed significantly higher levels of anxiety and depression than controls.	TMT-A & B
Dresler et al. 2012 [27]	Clinic-based	23	31	Combined group (MwA, MwoA) with no further subdivision.	Age range not reported	No	None specified	TMT-A & B
El-Senousy et al. 1995 [20]	Clinic-based	40	40	Combined MwA (12), MwoA (26), FHE (2), with ≤20 years of illness	Age range not reported	No	None specified	TMT-A & B
Gomez-Beldarrain et al. 2011 [21]	Clinic-based	84	41	Sub-group 1: Chronic migraine with drug overuse (42); Sub-group 2: episodic MwoA (42)	Sub-group 1: 41.21 (8.20); Sub-group 2: 36.19 (8.66); Controls: 37.12 (8.59)	No	Age ≤ 55 years; no past history of any neurologic disease different from migraine or a past history of any psychiatric disorder, except for depression or anxiety; no other chronic pain conditions; no general medical diseases; no psychotropic drugs.	TMT-B only
Hooker et al. 1986 [33]	Clinic-based	31	15	Sub-Group 1: MwA (16); Sub-Group 2: MwoA (15)	Sub-Group 1: 41.9 (14.9); sub-Group 2: 41.1 (17.1); Controls: 41.9 (14.3)	No	Migraine history ≥2 years; 1–10 attacks per months, each lasting ≥24 h; a maximum of one interval headache of grade 1 intensity/week allowed; no cluster headache; no muscle contraction headache; no history of central or peripheral nervous system disease or trauma, systemic disease, or major psychological disorder	TMT-A & B
Le Pira et al. 2014 [34]	Clinic-based	44	16	Sub-Group 1: MwA (12); Sub-Group 2: MwoA (32)	Sub-Group 1: 42.1 (10.2); Sub-Group 2: 36.7 (9.7); Controls: 35.8 (12.6)	No	No other types of headache, no history of central or peripheral nervous system disease, trauma, systemic diseases, major psychiatric disorder.	TMT-A & B
Lo Buono et al. 2017 [35]	Clinic-based	28	14	Sub-Group 1: MwA (14); Sub-Group 2: MwoA (14)	Sub-Group 1: 41.28 (13.44); Sub-Group 2: 40.75 (11.82); Controls: 41.75 (12.82)	Yes	Migraine history ≥10 years; no other types of headache; no vascular disease or trauma; no history of major psychiatric disorders; no metabolic disorders; no other neurological condition.	TMT-A & B
Martinez et al. 2010 [23]	Clinic-based	10	10	Migraine (sub-type not specified)	56.30 (6.83); Controls: 51.10 (6.70)	No	None specified but it was reported that migraine patients had no other neurological disorder.	TMT-A & B
Martins et al. 2012 [24]	Clinic-based	61	367	Migraine (sub-type not specified)	61.9 (7.6); Controls: 66.8 (9.0)	No	No known present or past history of a central nervous system disorder, including stroke, brain injury, epilepsy, dementia (known or suspected), psychosis, or a severe medical disorder like uncontrolled cancer, human immunodeficiency virus infection, renal or hepatic failure; mini mental state evaluation score above literacy-adjusted cutoff point	TMT-A & B
Tessitore et al. 2015 [28]	Clinic-based	40	24	Sub-Group 1: MwA (20); Sub-Group 2: MwoA (20)	Group 1: 30.10 (1.66); Group 2 30.05 (1.53); Controls: 29.15 (1.30)	No	No other type of headache, including chronic headache, somatic or psychiatric conditions, or intake of daily medication; patients were both aura and migraine free and not taking rescue medications for at least 3 day before testing.	TMT-A & B
Zeitlin & Oddy 1984 [36]	Clinic-based	19	19	Combined (MwA & MwoA) with no further subdivision	36.3; Controls 35.3 (range for all subjects: 20–50 years)	No	Migraine history ≥10 years; age < 51 years; Criteria by Crisp et al. (1977) for either common or classic migraine.	TMT-A & B

**Note.***FHE* Familiar Hemiplegic Migraine, *MwA* migraine with aura, *MwoA* migraine without aura, *SD* Standard Deviation

### Summary measures

The difference in mean number of seconds taken to complete each TMT section (TMT-A and TMT-B) between migraine patients and controls was used as the summary measure.

### Synthesis of results

Data were synthesized if at least 5 studies were included. Two initial meta-analyses were performed for TMT-A and B, separately, by collapsing together patients suffering from MwA and MwoA. These two subgroups were either already combined in the original studies or combined means and standard deviations were calculated. Specifically, in those studies in which different subgroups of migraine patients were compared with the same group of healthy controls, data from the different experimental groups were combined by using formulas reported in [37], to avoid unit-of-analysis error due to unaddressed correlation between the estimated intervention effects from multiple comparisons. Inconsistency was calculated as the percentage of total variation across studies due to heterogeneity (named I^2^), as it does not depend on the number of studies [38]. Cochran’s Q statistic was used as an additional measure of consistency/heterogeneity across studies.

### Risk of bias across studies

Risk of publication bias across studies was assessed through funnel plots [29]. In particular, if asymmetry was observed, the Trim-and-Fill method would impute potentially missing studies and adjust the combined effect size accordingly. The results of this approach should however be interpreted with caution, especially given that the included studies were few and not very homogeneous concerning several variables (age range, migraine duration, gender etc.).

### Additional analyses

Since MwoA is typically associated with more frequent and disabling attacks than MwA, additional meta-analyses were performed to appreciate the performance difference for these two types of migraine on TMT-A and B performance, limited to studies reporting these data separately. Five studies satisfied this criterion.

## Results

A PRISMA flowchart of the search and selection process is provided in Fig. 1.

### Characteristics of included studies

#### Methods

All 15 studies finally selected for the review were published in English. All the studies involved an evaluation of migraine patients and controls with a neuropsychological battery that included the TMT but also other tests. Some also included structural and/or functional neuroimaging evaluation [21, 26, 28, 34, 35] and psychiatric assessment [20], while another evaluated the effect of drug (over) use [25].

#### Participants

The included articles for the TMT-A involved 545 patients with migraine and 727 healthy controls, whereas, for the TMT-B, they included 629 patients with migraine and 768 healthy controls for the TMT-B. Commonly used inclusion criteria (see Table 1) entailed age ranges not involving children or older adults (with variable ranges in the adult lifespan across studies), length of history of migraine (> 1–10 years), a minimum number of attacks in the last year/month, normal brain neuroimaging, absence of other types of headache or chronic pain conditions, absence of other comorbidities (e.g., psychiatric, neurological, vascular or systemic diseases), no psychotropic drugs at time of testing, normal general cognitive function (e.g., no dementia; Intelligence Quotient, IQ > 80).

#### Intervention

Most of the works included in this review were focused on the assessment of neuropsychological deficits in migraine with TMT, versions A and/or B and other tests. Even those works which had other primary aims entailed some neuropsychological evaluation.

#### Outcome

The number of seconds to complete each section (TMT-A and B) was the chosen dependent variable. Other performance measures, such as errors, ratio (TMT-B/A) or difference (TMT B-A) scores, were excluded because they were very rarely reported. Separate effect sizes were calculated for each TMT part (A and B).

### Results of individual studies

**Fig. 2:**
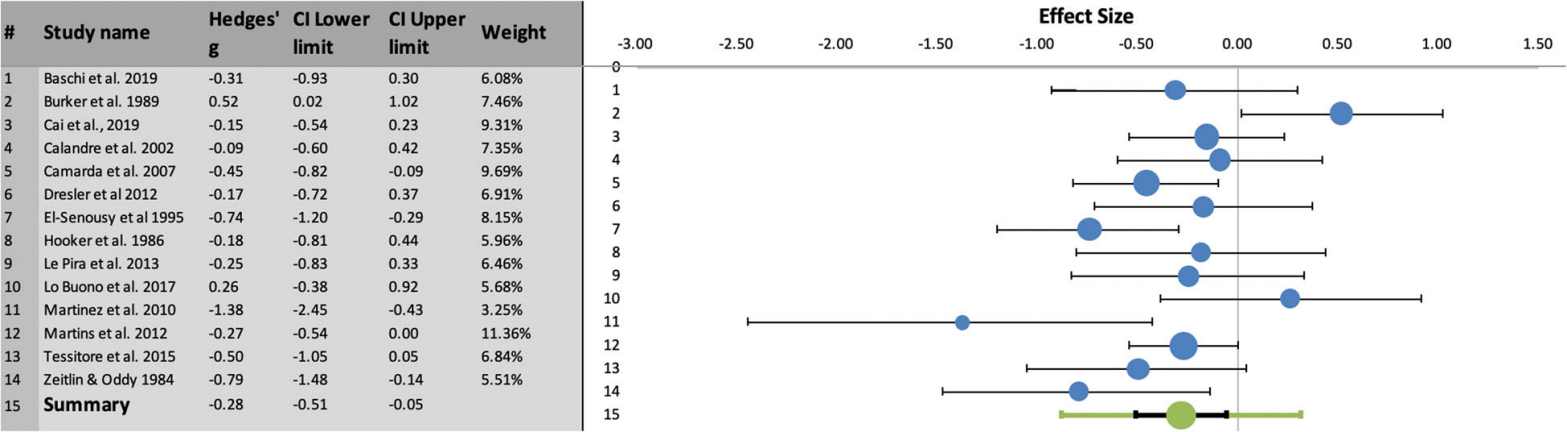
Left: Summary results of meta-analysis regarding TMT-A performance differences between migraine patients and healthy controls, including Hedges’ g, Confidence Intervals (CI) and relative weight of each study. Right: Forest plot showing the effect size (with confidence interval) of individual studies and, below, the combined effect size with its confidence interval (in black) and its prediction interval (in green)

TMT-A: In the pooled TMT-A analysis (Fig. 2), migraine patients performed significantly more poorly than healthy controls (Hedges’ g = −.28, SE = .11, 95% confidence intervals, CI = -.51/−.05, prediction intervals = −.88/.32; Z-value = − 2.66, two-tailed *p* = .008). There was moderate evidence of heterogeneity (I^2^ = 52.25%; Q = 27.23, p_q_ = .012).

**Fig. 3:**
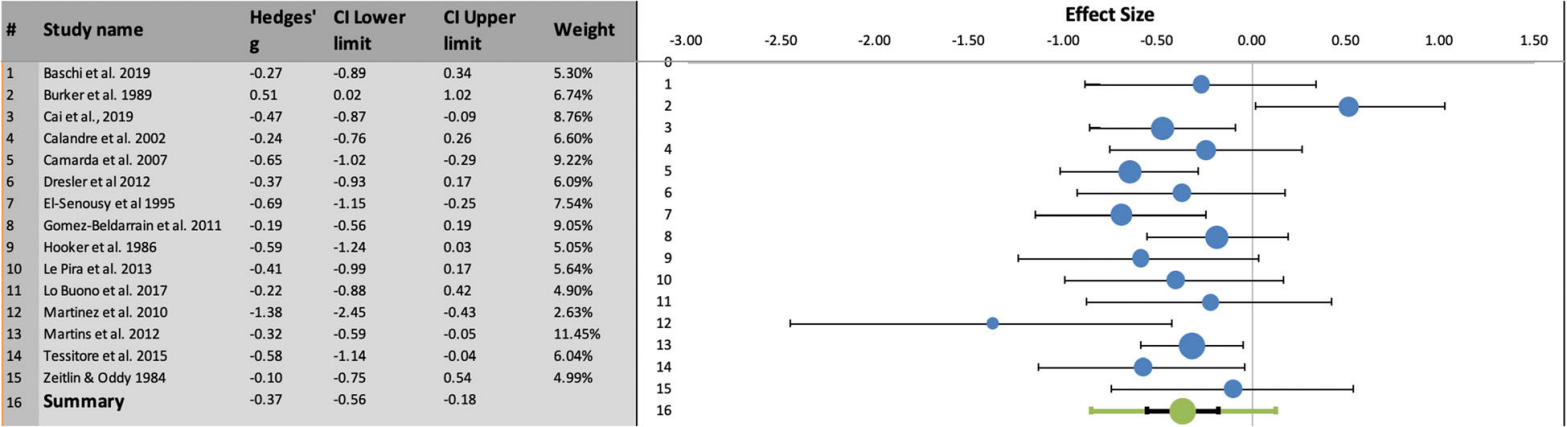
Left: Summary results of meta-analysis regarding TMT-B performance differences between migraine patients and healthy controls, including Hedges’ g, Confidence Intervals (CI) and relative weight of each study. Right: Forest plot showing the effect size (with confidence interval) of individual studies and, below, the combined effect size with its confidence interval (in black) and its prediction interval (in green)

TMT-B: In the pooled TMT-B analysis (Fig. 3), migraine patients performed significantly worse than healthy controls (Hedges’ g = −.37, SE = .09, 95% CI = -.56/−.18, prediction intervals = −.85/.12; Z-value = − 4.12, two-tailed *p* = .00004). There was modest evidence of heterogeneity (I^2^ = 43.33%; Q = 24.71, p_q_ = .038).

### Risk of bias across studies

**Fig. 4:**
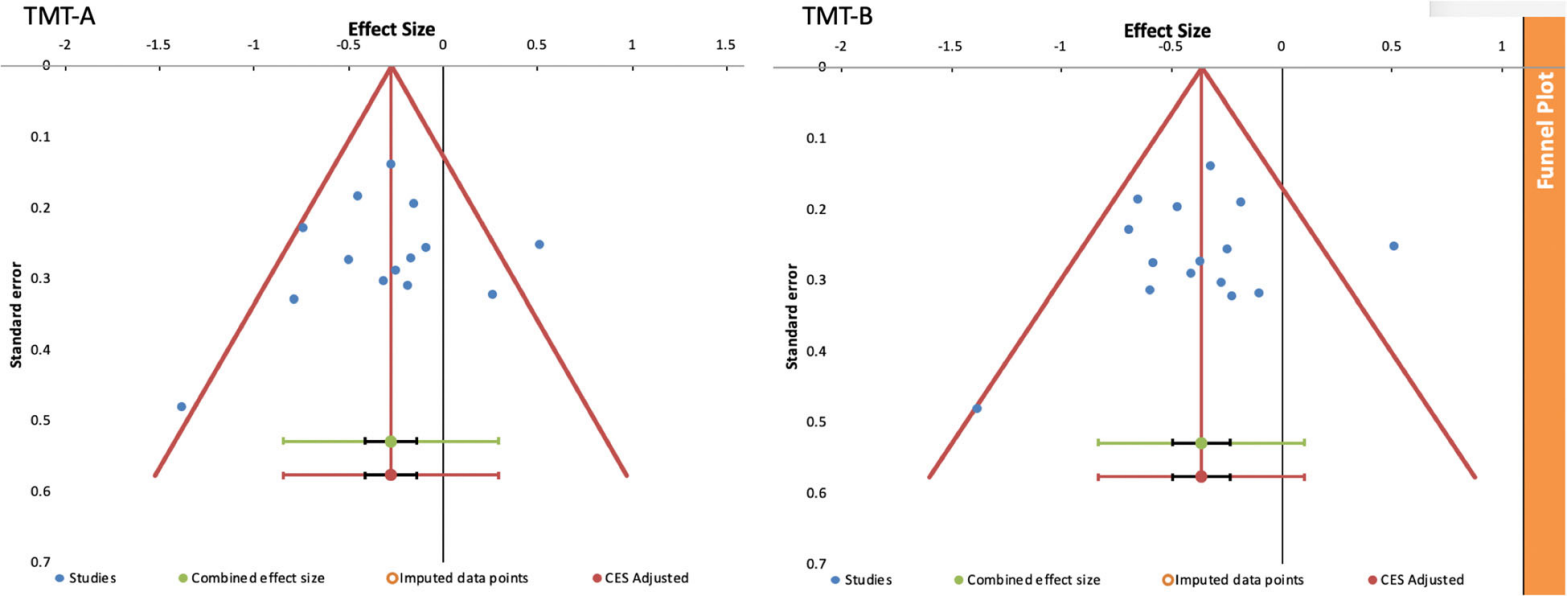
Funnel plots of the studies in the TMT-A (left) and TMT-B (right) meta-analyses (represented by blue dots), with effect size (on the x-axis above) and standard error (on the y-axis). The combined effect size (green dot) with its confidence interval (black) and prediction interval (green) is also shown. The plots also show a vertical line (in red) that runs through the (adjusted) combined effect size (CES) and the related lower and upper boundaries of the confidence interval (red diagonal lines). The absence of imputed data demonstrates no risk of bias

Modest evidence of heterogeneity was observed for both TMT-A (I^2^ = 52.25%; Q = 27.23, p_q_ = .012) and TMT-B (I^2^ = 43.33%; Q = 24.71, p_q_ = .038). However, funnel plots (Fig. 4) did not show any risk of bias across studies for either TMT-A or TMT-B, as no evidence of asymmetry was found.

### Additional analyses

**Fig. 5:**
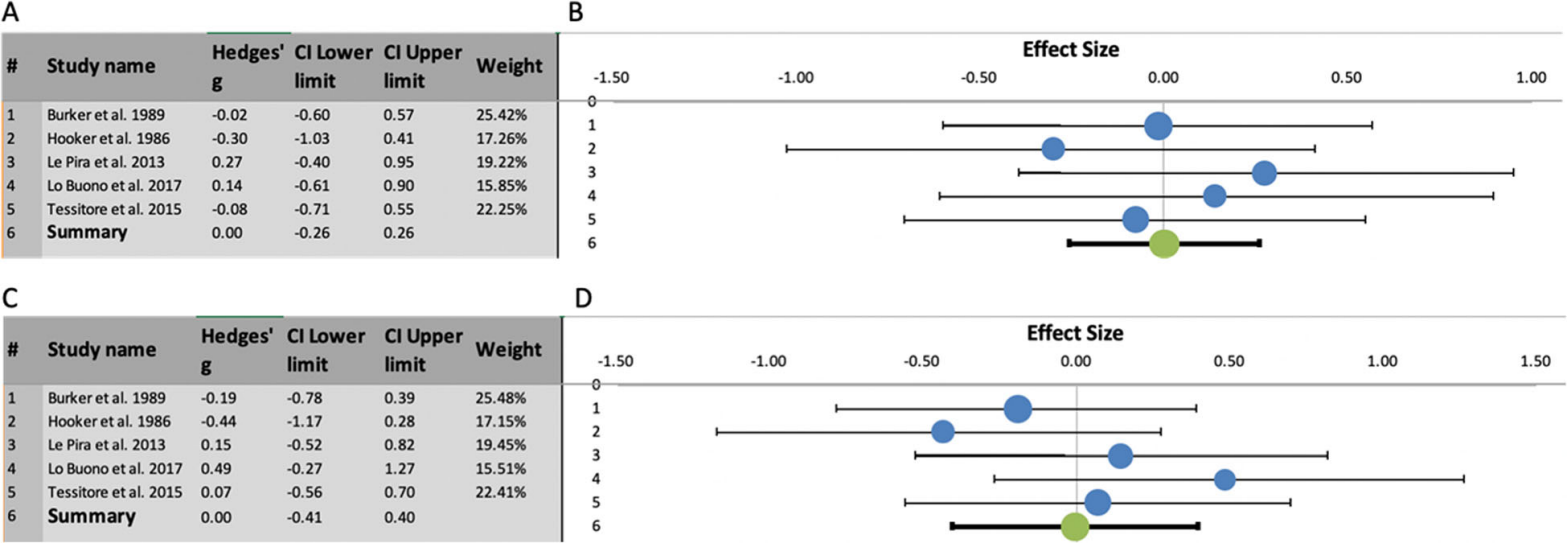
**a**: Summary results of meta-analysis regarding TMT-A performance differences between migraine with aura and migraine without aura, including Hedges’ g, Confidence Intervals (CI) and relative weight of each study. **b**: Forest plot showing the effect size (with CI) of individual studies and, below, the combined effect size with its CI (in black). **c**: Summary results of meta-analysis regarding TMT-B performance differences between migraine with aura and migraine without aura, including Hedges’ g, CI and relative weight of each study. **d**: Forest plot showing the effect size (with CI) of individual studies and, below, the combined effect size with its CI (in black)

In the meta-analyses contrasting migraine patients with and without aura (Fig. 5), there was no evidence of performance difference between these two groups either for TMT-A (Hedges’ g = 0, SE = .09, 95% CI = -.26/.26, prediction intervals = −.26/.26; Z-value = .01, two-tailed *p* = .992) or for TMT-B (Hedges’ g = 0, SE = .14, 95% CI = -.41/.40, prediction intervals = −.41/.40; Z-value = −.03, two-tailed *p* = .974). Moreover, there was no evidence of heterogeneity in the studies focusing on differences between MwA and MwoA either for TMT-A (I^2^ = 0%; Q = 1.60, p_q_ = .809) or for TMT-B (I^2^ = 0%; Q = 3.89, p_q_ = .421).

## Discussion

This meta-analytic study demonstrates that the TMT-A and B, which are used very frequently to assess cognitive abilities in migraine, are indeed useful neuropsychological tools to detect some of the cognitive deficits observed, even interictally, in patients suffering from MwA or MwoA. Specifically, the outcomes of the reported meta-analyses clearly showed migraine-related deficits in the amount of time necessary to complete both versions of the TMT.

Notably, the present analyses only focused on MwA and MwoA, and studies focusing on other types of migraine, and headache more generally, were not included. Thus, conclusions do not apply to othe important subsets of migraine patients. Additional meta-analyses evaluating potential performance differences between the specific subgroups of MwA and MwoA did not show any evidence of such differences either for the TMT-A or B. This is an important finding, as there is a debate in the literature concerning whether MwoA patients show a less severe cognitive impairment or even no disfunction when compared with MwA patients [8]. However, the interpretation of the latter ancillary analyses is limited by the fact that only few studies (*N* = 5) could be included.

It should also be noted that very few included studies adopted a blind design (*N* = 3). Moreover, some studies did not report important details, such as the migraine type, inclusion/exclusion criteria, gender composition or age range, which limits the generalizability of our findings and the possibility to carry out follow up meta-analyses regressing moderator variables on the effect size. Other studies were excluded because the reported information was not sufficient for the present purposes.

The TMT has reasonably high sensitivity, specificity and test-retest reliability from a clinical viewpoint [39, 40]; cf. [41]. However, the specificity of the cognitive constructs it measures is not high [42, 43]. Consequently, a derived score (e.g., TMT-B–TMT-A)/TMT-A), is recommended as a purer measure of executive functioning [17] which controls for general processing speed. However, derived TMT scores are rarely reported in studies on migraine. Moreover, while the results of this quantitative review clearly show that migraine is associated with cognitive deficits, only the performance on two tests (TMT-A and B) was taken into consideration. Some empirical studies failed to report cognitive deficits in migraine when other neuropsychological tests were used [3, 5]. Therefore, future work should also extend meta-analysis to other neuropsychological tests.

As a final remark, it would have been interesting to systematically evaluate the mediatory role of psychiatric comorbidities and treatment in the reported meta-analyses. There is indeed evidence that psychiatric comorbidities, such as depression and anxiety [44], and preventive medication [45, 46] may contribute to cognitive decline in migraineurs (see [47], for a review). However, the majority of studies comprised here either used these factors as exclusion criteria or did not mention them. Although it has been suggested that cognitive deficits in migraine are not fully explainable with prophylactic treatment and comorbidities [6], future meta-analyses should more carefully control for the impact of these factors when evaluating cognitive functioning in migraine patients.

## Conclusions

The present work tested the utility of the TMT, in its two main forms A and B, in detecting cognitive alterations in patients suffering from MwA and MwoA during the inter-ictal period. By using a meta-analytical approach, it was shown that the time needed to complete the TMT is generally longer in patients with migraine as compared to healthy controls, with no difference between the two migraine subcategories considered here (MwA and MwoA). These findings fully justify the recommendation that the TMT should be included in neuropsychological batteries aimed at evaluating the long-term impact of migraine on cognition and at monitoring treatment-related effects in this disease.

## Data Availability

Data and material used for this meta-analytical review can be shared, until two years after publication, upon reasonable request to the corresponding author from qualified researchers for purposes of replicating procedures and results.

## Abbreviations

FHE: Familiar Hemiplegic Migraine
IQ: Intelligence Quotient
MwA: Migraine with aura
MwoA: Migraine without aura
TMT: Trail Making Test
